# The Principles and Practice of Dental Surgery

**Published:** 1845-10

**Authors:** 


ART. VIII.—*The Principles and Practice of Dental Surgery.* By CHAPIN A. HARRIS, M.D., D.D.S., Professor of Practical Dentistry and Dental Pathology in the Baltimore College of Dental Surgery; Fellow of the American Society of Dental Surgeons; Member of the Medico-Chirurgical Faculty of Maryland, &c., &c. Second edition: revised, modified, and greatly enlarged. Illustrated by sixty-nine wood engravings. Philadelphia: Lindsay & Blakiston. 1845. 8vo. pp. 600.

This is a remarkably full treatise on the Teeth, and will doubtless become the manual of all dental surgeons in this country. The author has bestowed a very uncommon amount of labor upon it, with a view to rendering it as complete as any work could be in the present state of the art. It would be difficult, we suspect, to find any subject of interest to the dental surgeon which has been overlooked by the author, and a glance at the table of contents is sufficient to show that the maladies and deformities of the teeth, with their appropriate remedies, constitute an extensive and varied class of topics. The physician in the country will be able to make a profitable use of this volume, for he is often called upon to discharge the functions of the dentist. Many of the subjects are of a character to engage the attention of all classes of practitioners. Take the following as an example:

"*The Effects of Mercury, Tobacco, and Snuff upon the Teeth and Gums.*—Much has been said and written on the effects of these articles upon the dental organism, but there still exists much error of opinion on the subject, and for this reason, I cannot pass it by in silence.

"*Effects of Mercury.*—Mercury does not, as many imagine, exert any direct action on the teeth; it is only by the effects that it sometimes produces on the gums and secretions of the mouth, that they are injured by its use. When it is given in sufficient quantities, and its use continued long enough to produce ptyalism, however slight, it becomes hurtful to the teeth, and just in proportion as it affects the juices of the mouth, is the corrosive properties of these fluids increased. Hence, it can be considered only as an indirect cause of caries.

"The relation which the teeth sustain to the maxillæ, however, is often very seriously affected, and sometimes entirely destroyed by the exhibition of this medicine. Its introduction into the system is generally followed by an increased action of the glands' absorbents, and in no part of the body is this more evident than in the gums. It sometimes occasions a very rapid loss of substance in these parts, so that the teeth, by the destruction of their sockets, are loosened, and, in a few months, caused to drop out. A few years ago an application was made to me for a set of artificial teeth and gums, by an interesting young lady, of only twenty years of age, who had lost nearly the whole of her teeth, by the destruction of her gums and alveolar processes, caused by salivation. She informed me, that about four years before, she had been afflicted with a severe attack of bilious fever, and during its continuance, had taken a great deal of mercury, and, in consequence, had been so badly salivated, that her teeth were loosened, and soon after her recovery, one after another dropped out, in spite of the efforts of several physicians and dentists to preserve them, until only nine remained.

"The deposition of tartar upon the teeth, is much increased by the use of this medicine, especially when it affects the saliva. So much, in fact, is the tendency to a deposition of this substance, increased by its exhibition, that persons laboring, for the first time, under a mercurial diathesis, frequently have the crowns of their teeth, opposite the mouths of the salivary ducts, completely coated with it in a few days.

"In its administration, therefore, great care should be taken to prevent the injurious effects, that are liable to result from its use. Though, when given with proper precaution, it is perfectly safe in its action, yet, as a general rule, it should never be administered except by a person acquainted with the indications that it is expected to fulfil. It is not my purpose to discuss the merits of this article, but only to notice its effects upon the teeth, gums, &c.—yet at the same time, I cannot forbear deprecating the profuse and careless manner in which it is but too frequently given by persons unacquainted with the curative indications of those diseases for which it is usually prescribed. Its remedial virtues in many diseases, have been amply tested, so that its claims to confidence, rest on no doubtful foundation, and the most eloquent panegyric, that could be pronounced in its praise, is found in the hundreds of lives that it every year rescues from an untimely and premature grave. Its powerful and valuable medicinal properties have gained for it a justly deserved and high reputation; yet the popularity which it has thus justly acquired, has given to its use a licence replete with mischief. The imprudent manner in which it is frequently administered, during infancy and childhood, while the permanent teeth are being formed, cannot be too strongly censured. A mercurial action in the system, at these early periods of life, exerts a most deleterious influence on the physical structure of these organs, whereby their future liability to decay is greatly increased.

"The symptoms indicating a mercurial diathesis of the general system, are, a slight swelling of the tongue; soreness and increased redness of its edges; soreness, tumefaction, and preternatural redness of the gums, with a tendency to bleed from the slightest injury; fetor of the breath; viscid saliva, accompanied by a more copious discharge than usual; thickening of the alveolar periosteum, and loosening of the teeth. Such are the diagnostics that may be regarded as the criteria of the specific or constitutional action of the medicine.

"When given without proper care, and for any considerable length of time, it sometimes gives rise to sloughing and ulceration of the gums, and to necrosis and exfoliation of the alveolar processes, and portions of the jaw. Cases of this kind are of frequent occurrence. Several have come under my own observation within the last ten or twelve years; for the particulars of one of which the reader is referred to that of Mr. M., page 165. Ulcers of the gums, arising from an excessive use of mercury, sometimes assume a very malignant character, and are exceedingly difficult to cure.

"Some are more susceptible to the action of mercurial medicines than others. A single dose will, in some instances, produce ptyalism, while in others, a half dozen may be taken within a few hours of each other, without, in the least, affecting the secretions of the mouth.

"The gums, after having been affected with mercury, are ever after more susceptible to the action of irritants, and consequently more liable to become inflamed then they otherwise would have been. 'However perfectly,' says Mr. Bell, 'the effects of mercury may have subsided, even where no permanent injury appears to have been produced in the teeth, and where the common symptoms of its action have entirely ceased to exist, it is not at all an unfrequent circumstance, that after the lapse of a longer or shorter period, sometimes even several years, the teeth become loose, absorption of the gums and alveolar processes take place, and the early loss of the teeth is the consequence of an affection, the disappearance of which, for so long a time, had persuaded the patient that all danger of subsequent injury had ceased.'

"In conclusion, it is only necessary to observe, that, for the removal of the effects produced on the gums by the use of this medicine, a plan of treatment should adopted, similar to that previously recommended for spongy and inflamed gums. The offensive fetor of the breath may be corrected by frequently washing the mouth with diluted chloride of soda, or with the tinct. of myrrh.

"*Effects of Tobacco.*—In the supposed protective virtues of tobacco to the teeth, many find a ready excuse for its use. But its preservative qualities, if indeed it possesses any, have been greatly overrated. It is undoubtedly true, that, being a stimulant and narcotic, it will sometimes obtund the pain of an aching tooth; but even the relief thus obtained, is at best only temporary, and principally confined to those unaccustomed to its use; for those, who are in the daily habit of chewing or smoking, are as much subjected to toothache, as those who have never used it.

"As to the effects produced on the teeth themselves, by tobacco, I do not know that it matters much in what manner it is used, whether by chewing or smoking. Directly, it may be said to affect these organs, neither beneficially nor prejudicially. The increased flow of saliva which it occasions, may, perhaps, by diluting such vitiated humors, as happen to be in any of the interstices, or indentations of the teeth, and thus lessening their corrosive and acrid properties, render them less hurtful; yet this benefit is probably more than counterbalanced by its pernicious effects upon the gums. The constant state of excitement in which they are kept by its use, is apt, unless the greatest attention is paid to cleanliness of the teeth, to produce, especially in persons of a cachectic habit, a sort of chronic inflammation, and, in those of a strumous temperament, debility.

"In treating of the effects of this herb, Dr. Fitch observes: 'I have noticed persons having good teeth, who had used tobacco, both smoking and chewing it, for a great number of years. On the other hand, I have seen persons with very bad teeth, who used tobacco. In some constitutions, it removes its irritability; in others it increases the irritability of the system. And when we look over the causes of caries, diseased gums, &c., we shall find that the fact respecting the operation of tobacco, which we have noticed, explains its good and bad effects on different individuals. There is an acrimony or impurity about some tobacco, which causes it to injure the teeth; but my observations, as to the effect of good tobacco, are, that by some individuals it may be chewed with impunity, and that smoking tobacco may be allowed.'

"This article, as Dr. F. remarks, differently effects the general health of different individuals. While some can use it with impunity, its effects upon others are such, that many, even after having used it for a number of years, have been compelled to relinquish it entirely. A person of strong constitutional health, full habit, and of sanguino-bilious temperament, may employ it moderately without injury, and, in some cases, with advantage; but to one oppositely constituted, it is more or less productive of hurtful consequences. It is not, however, my design to treat of the constitutional action of this weed, but only to notice its effects on the teeth.

"From the coloring matter contained in tobacco, the teeth of persons, who chew large quantities of it, become, if great care be not taken to keep them clean, in a few years, so much stained, that they present a most filthy and disgusting appearance. Moreover, their teeth usually suffer a more than ordinarily rapid loss of substance, especially when the front ones fall plumb upon each other, from the constant abrasion to which they are thus necessarily subjected.

"Having thus briefly noticed the consequences that are likely to result to the teeth, from the use of this article, when chewed and smoked, I shall proceed to offer a few remarks on the manner in which it affects these organs when employed as a dentifrice.

"*Effects of Snuff.*—Within the last few years, snuff has, in some parts of the country, become quite popular as a dentifrice, especially with females. The teeth suffer more from the use of tobacco in this form, than in any other. Being reduced to a powder, its fine particles find a more easy lodgment beneath the edges of the gums, around the necks of the teeth, in their interstices, and various indentations and fissures, than when taken into the mouth in any other form. These particles not only thus serve as nuclei, around which the thickened and vitiated secretions of the mouth gather, but also, from their stimulating properties, and their long retention beneath the edges of the gums, and in the crevices of the teeth, are productive of much irritation, both to the gums and dental periosteum.

"I have observed, that the gums of persons, who have used snuff as a detifrice, for a length of time, usually have a dark purple, and sometimes a yellowish appearance, are soft and spongy, more or less isolated from the teeth, and that the teeth themselves are not unfrequently very much loosened. In fact, I do not recollect ever to have known an instance of an individual who had used tobacco in this way, two or three times a day, for several years, without the teeth and gums being thus affected. In some cases, however, it is much longer in producing these deleterious effects, than in others. Much depends upon the condition of the gums, at the time its use is commenced. If they be healthy, and firmly adhere to necks of the teeth, it may be employed for a considerable time, without being attended with any very obvious injury; but if they be at all diseased, a deleterious effect will soon become manifest. Viewing the subject in this light, and believing the opinion that I have here advanced, to be supported by the observation of every one, whose attention has been at all directed to the subject, I cannot but condemn the use of this article as a dentifrice; and recommend to every dentist, to particularly caution persons consulting him, and especially females, against thus employing an article teat is productive of such pernicious consequences to the teeth.

"Nor are its effects upon the general health less injurious. Persons who use snuff in this manner, are generally observed to have a pale, sallow countenance, especially if their constitutional health be at all delicate."

This extract affords a fair specimen of the style in which Dr. Harris has executed the several parts of his elaborate treatise. We take great pleasure in recommending it as a work of very high merit.

